# A Simple Surface Treatment for Improving the Adhesive Bonding Properties and Durability of an AlMg_3_ Alloy

**DOI:** 10.3390/molecules29245856

**Published:** 2024-12-12

**Authors:** Changfeng Fan, Bo Yang, Xue Wang, Xianghua Zhan, Xiaoli Yin, Jianmin Shi, Yancong Liu, Klaus Dilger

**Affiliations:** 1 College of Intelligent Manufacturing and Control Engineering, Shandong Institute of Petrochemical and Chemical Technology, Dongying 257061, China; 2 BYD Germany GmbH, Otto-Hahn-Street 21, 71069 Sindelfingen, Germany; 3 College of Chemical Engineering, Shandong Institute of Petrochemical and Chemical Technology, Dongying 257061, China; 4 College of Chemical Engineering, China University of Petroleum, Qingdao 266580, China; 5 Institute of Joining and Welding, Technische Universität Braunschweig, Langer Kamp 8, 38106 Braunschweig, Germany; 6 College of Mechanical and Electronic Engineering, China University of Petroleum, Qingdao 266580, China

**Keywords:** AlMg_3_ alloy, adhesive bonding, chemical treatment containing cerium, electrochemical measurement

## Abstract

The structural adhesive bonding of aluminum is widely used in the aircraft and automotive industries. The surface preparation of aluminum prior to adhesive bonding plays a significant role in improving the bonding strength. Surface cleanliness, surface roughness, and surface chemistry can be controlled, primarily, by proper surface treatment methods. In this study, the effect of varying the chemical treatment period on the adhesive bonding characteristics was investigated. An epoxy adhesive was used to join the treated surfaces, and the bond strengths were evaluated via single lap-shear (SLS) tests in pristine, as well as degraded, conditions. The surface morphology, chemistry, and corrosion properties of the surfaces with chemical treatments were characterized using various surface analytical tools, such as scanning electron microscopy, an energy dispersive spectrometer (SEM/EDX), and an electrochemical workstation. Excellent adhesion characteristics, with the complete cohesive failure of the adhesive, were encountered on the surfaces of the H_2_O_2_-treated samples. The H_2_O_2_-treated samples exhibited the highest initial bond strength, reaching 22.5 ± 0.5 MPa, and showed a decrease of only 10% (to 18.1 ± 0.2 MPa) after aging under extreme humidity and temperature conditions (70 °C and 100% R.H. for 4 weeks). The chemical treatment reported in this work is a very simple method to produce durable joints.

## 1. Introduction

The use of adhesive bonding in aluminum structures is prevalent in the aerospace, automotive, and marine industries because it offers several benefits compared to mechanical fastening or traditional methods like welding. These advantages include reduced corrosion and stress concentration, enhanced aesthetics, and cost-effectiveness [1,2,3,4]. Adhesive bonding facilitates large area connections and the joining of dissimilar materials with different thicknesses. Furthermore, it mitigates galvanic corrosion when bonding various metals due to the insulating properties of the adhesives. Adhesive bonding is typically lighter than mechanical fasteners and often requires minimal or no heat, thus eliminating the thermal distortion and residual stress commonly associated with heating [5].

The challenge for the industry is to identify an effective, straightforward, safe, and cost-efficient surface treatment method that results in strong bonding and long-term durability [6]. The surface pretreatment of aluminum alloys, especially for rolled and extruded types, has been recognized for a long time as a necessary step to guarantee both the initial bonding strength and the long-term durability of adhesive joints. The purpose of this procedure is to remove the mechanically weak natural surface oxide layer by substituting a new, uniform oxide layer, for which the bonding strength is increased [6,7].

As for aluminum surfaces, the pretreatment procedure usually consists of the modification of the surface by removing the native oxide layer and changing the microstructure and/or chemistry of the original material, which is the basis for the adhesive joining. Mechanical procedures are widely used for this purpose, which include sandblasting or grit blasting. The acid anodizing process confers an anodized layer on aluminum that boasts excellent corrosion resistance and robust adhesion strength, as referenced in [8]. The specific pretreatments of chromic acid anodizing (CAA) [9], phosphoric acid anodizing (PAA) [10], and sulfuric acid anodizing (SAA) [11] are known to enhance both the corrosion resistance and the bonding strength of aluminum alloys. However, the aluminum anodizing process is inherently water-intensive, generating substantial waste that is typically managed through sewage systems or landfill disposal, often after some form of treatment. The waste, predominantly composed of aluminum hydroxide, sodium or calcium salts, and aluminum sulfates, contains up to 85% water by weight [12]. Chemical treatments represent an efficient and cost-effective approach for preparing aluminum alloys for adhesive bonding, making them widely utilized in industry [8,13]. This process usually consists of a number of processes, such as the deposition of oxide coatings by chemical conversion baths, deoxidation in acidic solutions, and degreasing in alkaline solutions. Ti- and/or Zr-oxides, Ce-oxides, Cr-oxides, silicates, and phosphates have been the focus of regulatory efforts as a result of the environmental and health concerns that surround chromate conversion coatings containing hexavalent chromium [14,15,16,17].

Many industrial companies utilize chemical pretreatment processes for various aluminum alloys without adapting the procedures specifically for AlMg_3_ alloys, which may result in ineffective surface preparation. According to some research, epoxy-bonded aluminum pretreatment with Zr-based conversion coatings shows similar performance as that of aluminum treated with chromate-phosphate [18]. Lunder et al. showed that Zr–Ti-oxide conversion coatings offer enhanced durability for aluminum alloys bonded with adhesives [19]. Surface passivation and better corrosion resistance are the reason that Zr/Ti-oxide-coated aluminum alloys were improved in corrosive conditions due to the increased durability of the alloy. Among the alternatives for corrosion protection, rare-earth coatings, particularly those based on cerium, have gained significant attention in relation to epoxy coatings [20], magnesium [21,22], and aluminum [23,24]. Cerium exhibits a significant ability to form a stable oxide and hydroxide film, which not only enhances its functional applications but also emphasizes its non-toxic characteristics and cost-effectiveness [25]. In a detailed study, M. Kanani and Johansen et al. examined the combined synergistic effects of cerium conversion, particularly the use of a conducting polymer duplex coating, in order to increase the corrosion resistance of an AA6063 aluminum alloy. Their results demonstrate the potential of this innovative approach to substantially enhance the durability and longevity of aluminum components subjected to corrosive environments [19,26]. The cerium-based film produces an effective corrosion-resistant layer and reliable bonding characteristics for the adhesive bonding on magnesium alloys [27] and the 1050-H14 aluminum alloy [28]. Few studies focus on the cerium-based chemical coatings and their role in providing interfacial strength and reliable durability in adhesive bonding on an AlMg_3_ alloy.

In this study, a straightforward technique for eliminating the weak native oxide layer while simultaneously roughening the surface by immersing aluminum substrates in a specific solution was introduced. The adhesive bond strength of these surfaces and their durability in extreme humidity and temperature conditions were examined. A comparison of the performance of various chemical treatment procedures was reported in this paper, which focused on changes in the surface morphology and chemical composition both before and after pretreatment. Additionally, corrosion properties were evaluated through electrochemical measurements, and adhesion characteristics are assessed using single lap-shear tests, both with and without aging.

## 2. Experimental Section

### 2.1. Materials and Chemical Treatment

The material applied was a commercial AlMg_3_ aluminum alloy, supplied by Shanghai Miandi Metal Group Co., Ltd. (Shanghai, China). The chemical composition is listed in Table 1. A plate with the substrate thickness of 1.5 mm was cut into the size of 100 mm × 25 mm.

**Figure 1:**
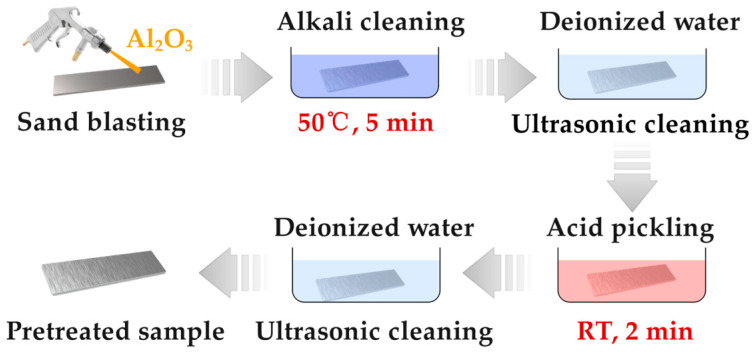
AlMg_3_ Surface preparation prior to chemical treatment.

**Figure 2:**
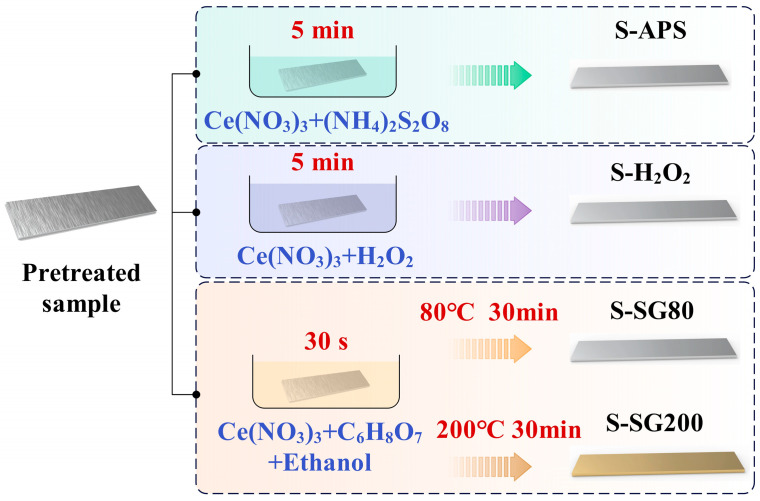
Different chemical treatments (APS, H_2_O_2_, SG80, SG200) on the pretreated AlMg_3_ surface.

Figure 1 and Figure 2 illustrate the chemical treatment process that includes degreasing, deoxidation, and the application of chemical coatings. The AlMg_3_ samples were first subjected to sandblasting, followed by immersion in an alkaline degreasing solution containing sodium hydroxide and surfactant additives. Crushed aluminum oxide was selected as the sandblasting medium, a prevalent and potent abrasive for metal surface preparation. The feeding pressure of the medium was meticulously set at 8 bar, a standard that ensures efficient surface roughening while preventing undue substrate damage. The nozzle was consistently held at a distance of 10 cm from the AlMg_3_ alloy’s surface to guarantee a uniform abrasion effect. The nozzle angle was optimized at 45° to the horizontal surface, facilitating a uniform distribution of abrasive particles across the surface. The sandblasting duration was standardized at 60 s per sample, a duration found through preliminary tests to be sufficient for achieving the targeted surface roughness without causing material overheating. Following rinsing with tap water and ultrasonic cleaning with deionized water, some of the degreased samples were subjected to deoxidation in a sulfuric acid solution at room temperature for 2 min. The ultrasonic cleaning process was executed at 400 W and a frequency of 40 kHz in deionized water. Each sample was subjected to 10 min ultrasonic cleaning. The cleaning solution’s temperature was controlled to be below 40 °C. Following deoxidation and another round of ultrasonic cleaning with deionized water, the samples were then coated at room temperature with three different chemical solutions [29,30,31,32]: an APS solution (0.1 M Ce(NO_3_)_3_ + 0.1 M (NH_4_)_2_S_2_O_8_ + Ethanol), an H_2_O_2_ solution (0.1 M Ce(NO_3_)_3_ + 0.1 M H_2_O_2_ + Ethanol), and a sol-gel solution (0.1 M Ce(NO_3_)_3_ + 0.1 M C_6_H_8_O_7_ + Ethanol) for 5 min, 5 min, and 30 s, respectively [33,34]. Finally, the chemically treated samples (designated as S-SG80) were dried at 80 °C for 30 min, while one group of samples, treated with the sol-gel solution (denoted as S-SG200), was dried at 200 °C for 30 min. The samples treated with the APS, H_2_O_2_, and sol-gel solutions are referred to as S-APS, S-H_2_O_2_, S-SG80, and S-SG200, respectively. Each schematic represents the immersion of the deoxidized samples in their respective solutions. The figures show the chemical composition of the solutions and the expected reaction on the sample surface. The APS and H_2_O_2_ solutions are designed to form a cerium-based chemical coating, while the sol-gel solution is intended to create a more durable coating through a controlled drying and densification process.

### 2.2. Single Lap-Shear Samples

**Figure 3:**
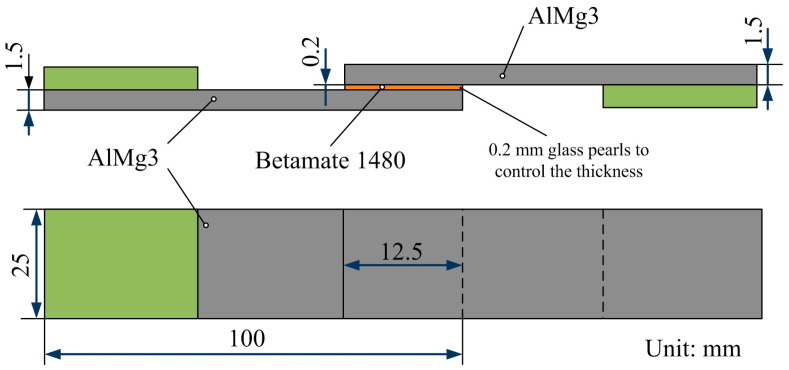
The single lap-shear joint of AlMg_3_ with Betamate 1480 epoxy adhesive.

Single lap-shear joints, as illustrated in Figure 3, were prepared in accordance with DIN EN 1465 [35]. The samples, measuring 100 mm × 25 mm × 1.5 mm, were either in the as-received or the chemically treated condition. With the use of Betamate 1480 (Dow Corning, Wiesbaden, Germany), the samples were bonded with an overlap length of 12.5 mm in either the as-received or the chemically treated condition, with the exception of one component that was commonly used in automotive body structures. The adhesive was adjusted by dispersing several glass pearls with a diameter of 0.2 mm inside the bondline. The adherents were fixed, and the adhesive was applied. The joints were cured in a laboratory furnace at 180 °C for 30 min after the adherents had been fixed. For every group, at least three samples were made in both the as-received and the chemically treated states. The single lap-shear samples were tested before and after aging (at 70 °C and 100% relative humidity for 4 weeks) under ambient conditions (25 °C and 50% relative humidity) at a constant displacement rate of 1 mm/min using an Instron 5567 testing machine(INSTRON, Norwood, MA, USA). The test procedure was managed and the data were logged using Bluehill software (Version 3.13). According to the load versus displacement curve, the single lap-shear strength was computed using the maximum load recorded in the curve.

### 2.3. Electrochemical Measurement

Electrochemical measurements were conducted in a 3.5 wt.% NaCl solution using an electrochemical workstation (Princeton Applied Research, Oak Ridge, TN, USA, VersaSTAT4). A silver/silver chloride electrode (Ag/AgCl, 3 M KCl) was employed as the reference electrode, while a platinum mesh served as the counter electrode. The working electrode was secured to a flat cell with a 1.0 cm^2^ exposure area to the electrolyte. The open circuit potential (OCP) was measured for 900 s to characterize the electrochemical response of the samples and ensure that the system reached a stable state. The measurements were carried out in dynamic polarization at a scanning rate of 1 mV/s, which was changed by 0.1 V in comparison to the OCP. Electrochemical impedance spectroscopy was used to perform the measurements at the OCP, which was operated at a frequency range of 100 kHz to 10 mHz, with a 20 mV signal amplitude. ZView software (Version 3.1, Scribner Associates, Inc., Southern Pines, NC, USA) was used to fit and analyze the measurement results with a specified equivalent circuit.

**Figure 4:**
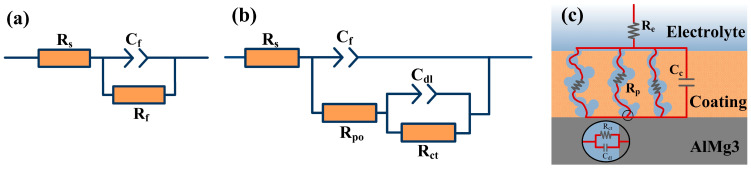
The equivalent circuit of model A (**a**) and model B (**b**) used to fit the EIS, and the physical representation of the cerium-based chemical coating exposed to the electrolyte (**c**).

Model A, illustrated in Figure 4a, was proposed to interpret the electrochemical impedance spectroscopy (EIS) spectra in the stage prior to the electrochemical processes [36]. The solution resistance can be regarded as R_s_. For the bare alloy, only one layer existed that can be associated with the elements (oxide film capacitance C_f_ and oxide film resistance R_f_). Constant phase elements (CPEs) are added to get more precise fitting results, which take into account the system’s non-ideal capacitive behavior of C_c_ and C_dl_. The following is the expression for this:

(1) $$Z\left(j\omega \right)={\left(Y_{0}\right)}^{-1}{\left(j\omega \right)}^{-n}$$

*Y*_0_ denotes the constant phase element, measured in F·cm^−2^s^n−1^, where *j* signifies the imaginary unit, *ω* represents the angular frequency, and *n* indicates the degree of deviation from purely capacitive behavior, with a range of 0 ≤ *n* ≤ 1. The capacitance approaches the *Y*_0_ of the CPE when *n* is approximately 1. For the samples with chemical coatings, the capacitance, C_f_, can be interpreted as the capacitance of the chemical coating. Along with the exposure time, the chemical coating gradually weakens, leading to a continuous increase in pore formation, resulting in a roughly homogeneous distribution of electrochemical reactions. Herein, R_p_ is related to the pores and microcracks on the chemical coatings. Model B, shown in Figure 4b, was used to explain the electrochemical reaction. This electrochemical reaction is represented as a double-layer capacitance(C_dl_) in the form of a charge transfer resistance(R_ct_) in parallel with the electrochemical component, as shown in Figure 4c.

## 3. Results and Discussion

**Figure 5:**
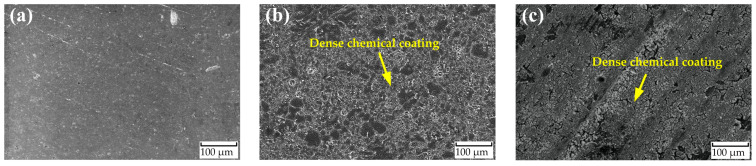
SEM micrographs for AlMg_3_ samples: (**a**) as received, (**b**) with APS solution treatment, and (**c**) with H_2_O_2_ solution treatment.

The surface morphologies of the samples of AlMg_3_ substrate, S-APS, and S-H_2_O_2_ are shown in Figure 5. The uncoated sample depicted in Figure 5a displays many irregularities, even though its surface was polished with silicon carbide paper up to grade #2400. The scanning electron microscopy (SEM) micrographs depicted in Figure 5b,c reveal the formation of a dense and porous protective film composed of cerium agglomerates, which covers nearly the entire surface. This coating seems homogeneous and uniformly deposited on the AlMg_3_ substrate. At the cathodic sites created by the intermetallic compounds inside the aluminum matrix, cerium deposits start to develop. The reduction of hydrogen peroxide results in a localized rise in pH, which facilitates this growth. Additionally, the solution’s elevated pH values (approximately 5.5 to 4) encourage the creation of more hydroxyl ions [37,38]. The accumulation of cerium deposits effectively obstructs the cathodic zones, leading to a decrease in the system’s cathodic current and improving the corrosion protection performance. The coating deposition was performed through an ammonium persulfate- or hydrogen peroxide-assisted treatment. The varying coverage of cerium oxides on the surface could be linked to differences in reactivity, as noted and discussed in relation to the chemical pretreatment of the aluminum alloys.

**Figure 6:**
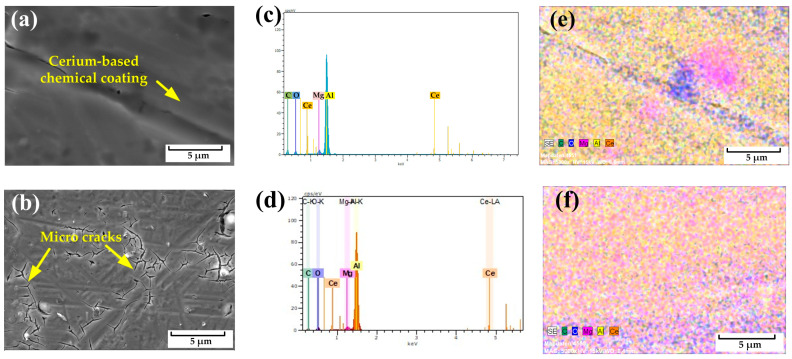
SEM micrographs for the AlMg_3_ samples S-SG80 (**a**) and S-SG200 (**b**); the energy-dispersive X-ray spectra of S-SG80 (**c**) and S-SG200 (**d**); and the element distribution of S-SG80 (**e**) and S-SG200 (**f**).

Figure 6a,b show the scanning electron microscope surface morphology of the cerium oxide films on the S-SG80 and S-SG200 samples, which was used to verify the precipitation of cerium chemical coatings. It can be observed that the films fully cover the surface of the AlMg_3_ substrate. The only problem arising from making a dense sol-gel coating is that the shrinkage caused by drying out the sample produces tiny cracks [39]. These microcracks are associated with thermal treatment during the drying process, where the evaporation of water from the coating leads to shrinkage and subsequent cracking. Sol-gel coating formation parameters, like temperature, curing, crack formation during and heat treatment, etc., are of significant importance and need to be studied to gain a more thorough understanding of the process and the control of the coating properties [31]. The S-SG200 sample, treated at 200 °C for 30 min, displays a greater density of microcracks compared to the S-SG80 sample [40]. As the coating dries, it undergoes shrinkage, which generates stress that can lead to the development of microcracks [41]. These microcracks not only compromise the integrity of the coating but also serve as initiation points for further crack propagation, ultimately affecting the adhesion bonding strength of the material. Corrosive media can more easily pass through these microcracks to reach the substrate when the coating is exposed to an electrolyte for prolonged periods of time. Interfacial corrosion results from this penetration, which weakens the adhesive bonding even more. The corresponding energy-dispersive X-ray (EDX) spectra collected from these surface regions are shown in Figure 6c,d, depicting peaks that correspond to the presence of cerium. The distribution of the elements on the surfaces of the S-SG80 and S-SG200 samples is illustrated in the EDX mappings in Figure 6e and Figure 6f, respectively. From the distribution patterns, it can be inferred that the coating consists of Ce-oxide, Al-oxide, and Mg-oxide.

**Figure 7:**
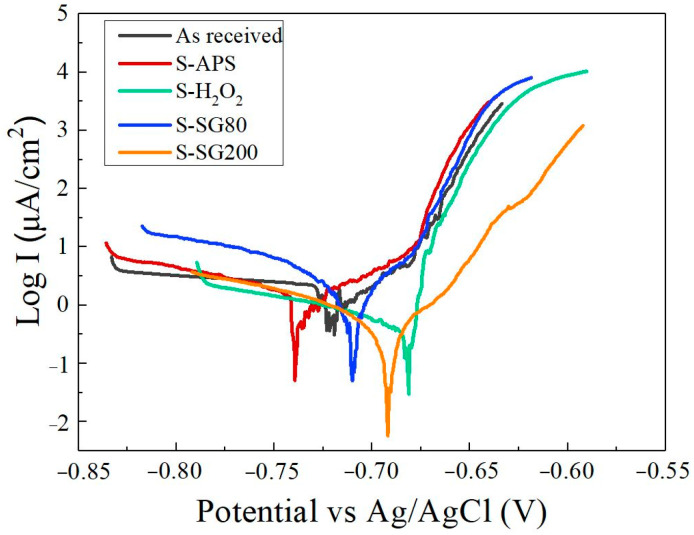
Potentiodynamic polarization curves in 3.5 wt.% NaCl solution on AlMg_3_ and AlMg_3_ treated with different chemical treatments.

Figure 7 presents the potentiodynamic polarization curves of the AlMg_3_ samples subjected to different treatments in a 3.5 wt.% NaCl solution at room temperature. The polarization behavior of the samples is similar, indicating that the electrochemical response of the AlMg_3_ alloys is predominantly influenced by cerium ions. The curves demonstrate that the corrosion potential of the alloys is approximately −0.70 V (Ag/AgCl). The polarization curves for both the as-received alloys and the treated samples (S-H_2_O_2_ and S-SG200) demonstrate a decrease in cathodic and anodic currents by nearly two orders of magnitude, with the S-SG200 sample showing the lowest corrosion current density (*i*_corr_ = 0.25 μA/cm^2^). The cerium oxide layer, which acts as an inhibitor by preventing the cathodic reaction and, as a result, the rate at which the dissolution occurs, can be used to explain this behavior. High temperatures and the presence of hydrogen peroxide and ammonium persulfate promote the development of cerium oxide/hydroxide films, as shown by the reactions below [42,43]:

(2) $$O_{2}+2H_{2}O+4e^{-}\to 4{OH}^{-}$$

(3) $${\left({NH}_{4}\right)}_{2}S_{2}O_{8}+2H_{2}O\to {2NH}_{4}H{SO}_{4}+H_{2}O_{2}$$

(4) $$H_{2}O_{2}+2e^{-}\to 2{OH}^{-}$$

The reaction can be used to produce the cerium oxide/hydroxide layer formation, which is as follows:

(5) $${Ce}^{3+}+{OH}^{-}\to Ce{\left(OH\right)}_{3}\to {Ce}_{2}O_{3}$$

Considering the data obtained for the samples with different treatments, the higher current densities observed in the samples with chemical treatment layers and sol-gel films can be related to the lower coverage of the uncovered active areas on the alloy, which results in increased metal dissolution. The S-SG200 samples, however, exhibit a more pronounced recovery, demonstrating their beneficial effect. These features can be confirmed with the SEM analysis.

**Figure 8:**
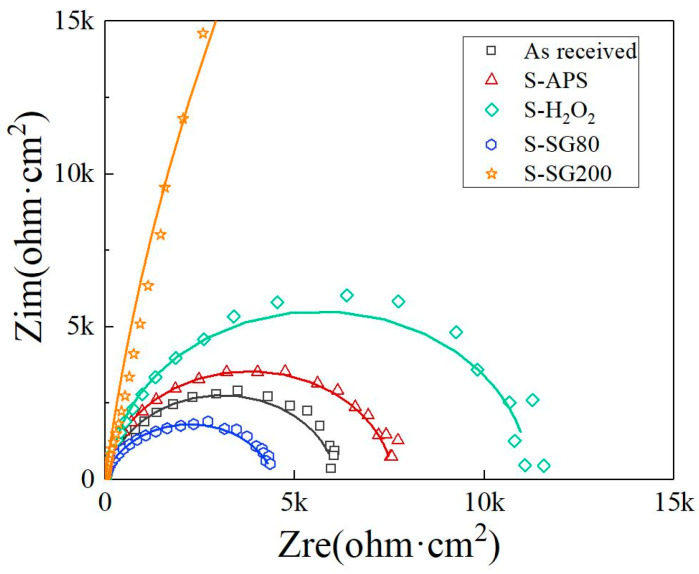
EIS Nyquist plots in 3.5 wt.% NaCl solution on AlMg_3_ and AlMg_3_ treated with different chemical treatments.

EIS measurements were conducted to characterize both the substrate and the treated samples. The samples treated with cerium chemical coatings and sol-gel films and the Nyquist plots for the AlMg_3_ alloy are shown in Figure 8. It can be observed that, for the S-SG200 sample, only one clear capacitive loop appears in the Nyquist plot, which can be attributed to the sol-gel film. Generally, R_pl_ (R_pl_ = R_po_ + R_ct_) is inversely proportional to the corrosion rate of the alloy and is commonly used for evaluating corrosion behavior. In contrast, the EIS plots for the other samples exhibit a diminished capacitive semicircle throughout the entire frequency range. Two capacitive loops are observed in these Nyquist plots: one at higher frequencies, associated with the oxide layer, and another at lower frequencies, related to the interfacial reaction. The capacitance values associated with the porous oxide layer and the electrochemical reaction are replaced by the constant phase elements Q_c_ and Q_dl_, respectively. The modification is necessary when the phase angle of the capacitor deviates from −90°. The results obtained from the EIS fitting are highly consistent with the potentiodynamic polarization in Figure 7. The S-SG200 and S-H_2_O_2_ samples exhibited the highest impedance values, measuring 203.9 kohm·cm^2^ and 11.2 kohm·cm^2^, respectively, indicating superior corrosion resistance. In contrast, the S-SG80 sample, with a resistance of 4.4 kohm·cm^2^, demonstrated comparatively lower anticorrosion properties.

**Figure 9:**
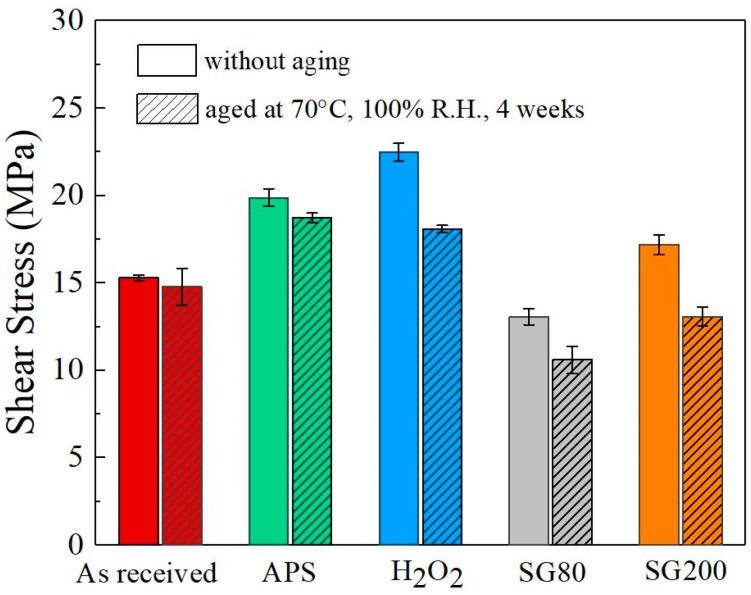
Adhesion strength measured by single lap-shear tests on the samples treated with different chemical treatments with/without aging at 70 °C and 100% relative humidity for 4 weeks.

Single lap-shear tests were conducted on the samples with various surface treatments. The shear strength of the samples treated with different chemical layers and films is displayed in Figure 9, with comparisons to the as-received sample. The as-received sample showed a combination of interfacial and cohesive ruptures, yielding a shear strength of approximately 15.3 ± 0.2 MPa under pristine conditions. For the samples treated with the APS, H_2_O_2_, SG80, and SG200 solutions, the measured strengths were approximately 19.9 ± 0.5 MPa, 22.5 ± 0.5 MPa, 13.1 ± 0.5 MPa, and 17.2 ± 0.5 MPa, respectively, in the unaged state. The mode of failure for the chemically treated surfaces was predominantly cohesive, indicating stronger adhesion properties as a result of surface treatment. Notably, the sample treated with the H_2_O_2_ solution exhibited the highest bond strength and a completely cohesive failure mode, indicating a superior adhesion performance. Aging at 70 °C and 100% relative humidity for 4 weeks caused a reduction in the shear strength across all samples, with the treated samples retaining relatively higher bond strengths compared to the untreated control. After aging, the shear strengths decreased to approximately 14.8 ± 1.1 MPa for the as-received sample, 18.7 ± 0.3 MPa for the APS-treated sample, 18.1 ± 0.2 MPa for the H_2_O_2_-treated sample, 10.6 ± 0.8 MPa for the SG80-treated sample, and 13.1 ± 0.6 MPa for the SG200-treated sample. The data demonstrate that the H_2_O_2_-treated sample maintained the highest bond strength even after aging, suggesting that this treatment provides enhanced durability and resistance to environmental degradation. The oxidative action of hydrogen peroxide on the aluminum surface, which results in the formation of a more-dense chemical coating, is responsible for the improved performance of the H_2_O_2_-treated samples. This layer serves as a barrier against environmental factors, including moisture that, over time, may weaken the strength of the bond. Furthermore, the H_2_O_2_ treatment provides the ideal surface conditions for adhesion, as seen by its ability to maintain a high bond strength in both pristine and aged conditions. One approach which exhibits promise for enhancing the performance and durability of adhesive bonding in aluminum alloys in industrial settings is the H_2_O_2_ treatment. It has the ability to improve adhesive joints’ durability in a variety of environmental conditions. The significance of surface preparation for achieving strong and durable adhesive bonding will be emphasized by this analysis.

**Figure 10:**
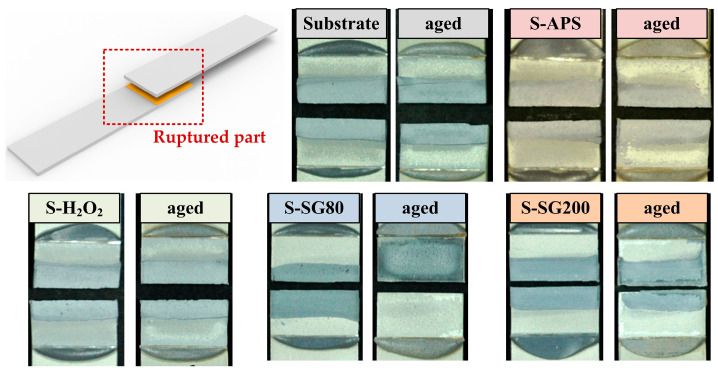
Images of the ruptured samples treated with different chemical treatments with/without aging at 70 °C and 100% relative humidity for 4 weeks.

The lap-shear strengths measured on chemically treated surfaces are similar to those attained through various anodizing processes, which are commonly considered standard surface treatments in adhesive bonding. Zhang et al. reported adhesion strengths of ca. 23 MPa for aluminum surfaces anodized with phosphoric and chromic acids and approximately 20 MPa for those anodized with boric and sulfuric acids [44]. Anodization involves the use of strong acids and several steps, including pretreatment to eliminate the native oxide layer and post-treatment to seal the anodized pores. In contrast, this study demonstrated that single-step chemical treatments produced adhesion strengths similar to those found in anodized surfaces. Figure 10 displays images of the fractured samples that were treated with chemical solutions (S-APS, S-H_2_O_2_, S-SG80, S-SG200) alongside a sample that underwent sandblasting and acetone degreasing.

The S-APS and S-H_2_O_2_ samples, which demonstrated full cohesive failure during the single lap-shear tests, were subsequently assessed for environmental durability by subjecting the adhesively bonded samples to extreme temperature and humidity conditions (70 °C, 100% R.H.) for four weeks. Figure 9 displays the lap-shear strength data for these samples. After degradation, the S-APS and S-H_2_O_2_ samples retained their cohesive failure mode despite harsh environmental exposure, indicating strong environmental stability. The current study demonstrates that chemical treatments provide a simpler alternative to complex anodization processes, which often involve hazardous acids like sulfuric, phosphoric, and chromic acids and multiple pre- and post-treatment steps. The S-SG80 and S-SG200 samples, which exhibited high anticorrosion properties (electrochemical impedance values of approximately 203.9 kohm·cm^2^ and 11.2 kohm·cm^2^, respectively), primarily showed adhesive failure. The cerium coating synthesized via the sol-gel process demonstrated inferior adhesion characteristics [45]. Despite the fact that this coating offers commendable corrosion resistance, it does not constitute an ideal solution that harmoniously combines both adhesion properties and outstanding corrosion resistance. Herein, this failure mode can be attributed to weak interfacial bonding between the sol-gel coating and the AlMg_3_ substrate. To guarantee improved interfacial performance, future considerations might entail using other surface treatment techniques. However, it might be possible to create sol-gel coatings with a stronger binding by using different methods [46].

The chemical treatment method plays a critical role in enhancing adhesion performance on aluminum surfaces, both under pristine conditions and after environmental exposure. Hydrogen peroxide acts as an accelerator in the oxide formation process, and this improvement can be explained by the production of cerium oxides. The standard reaction is driven by an increase in pH, which results from the reduction of oxygen and is further promoted by metal oxidation. Ce^4+^ is favored to reduce to Ce^3+^ under acidic circumstances because cerium is more stable at lower pH values, as shown by the Pourbaix diagram. Conversely, Ce^4+^ becomes more stable at higher pH values, especially in the presence of oxidizing agents such as O_2_ or H_2_O_2_ [26].

When hydrogen peroxide is introduced by the reaction described above or as an H_2_O_2_ solution, the oxide formation reaction is accelerated because of the creation of hydroxyl ions from the direct reduction of hydrogen peroxide. This process enhances the formation of protective cerium oxide layers on the aluminum surface, thereby improving the adhesion strength and durability of the treated joints.

(6) $${Ce}^{3+}+{OH}^{-}+\frac{1}{2}H_{2}O_{2}\to Ce{\left(OH\right)}_{2}^{2+}$$

(7) $$Ce{\left(OH\right)}_{2}^{2+}+{2OH}^{-}\to {Ce\left(OH\right)}_{4}$$

(8) $${Ce\left(OH\right)}_{4}\to {CeO}_{2}+2H_{2}O$$

At a high concentration of OH^−^, Ce^3+^ can be oxidized by dissolved oxygen or hydrogen peroxide to Ce^4+^, according to reaction (9) or (10).

(9) $${4Ce}^{3+}+O_{2}+{4OH}^{-}+2H_{2}O\to 4Ce{\left(OH\right)}_{2}^{2+}$$

(10) $${2Ce}^{3+}+{2OH}^{-}+H_{2}O_{2}\to 2Ce{\left(OH\right)}_{2}^{2+}$$

It has long been known that in coatings, cerium usually remains as Ce^3+^ or is oxidized to Ce^4+^ in the presence of condensed moisture and atmospheric oxygen. Ce^3+^ ions can transform into Ce^4+^ through the formation of cerium hydroxides and oxides at cathodic sites, which are induced by a local increase in pH. The cerium ions in a solution can come into contact with decreasing conditions when an inorganic coating is applied. The bare metal that is exposed by a coating defect permits Ce^4+^ to revert back to Ce^3+^, which, in turn, reduces the solubility of cerium. This dynamic redox process enables the coating to release cerium ions that can then re-precipitate, potentially self-healing minor defects in the coating. The advantage of the currently reported method lies in its ability to eliminate multiple steps in the surface treatment process. This simplified approach provides a significant improvement in the environmental durability of adhesively bonded joints by applying chemical treatments through a straightforward procedure.

**Figure 11:**
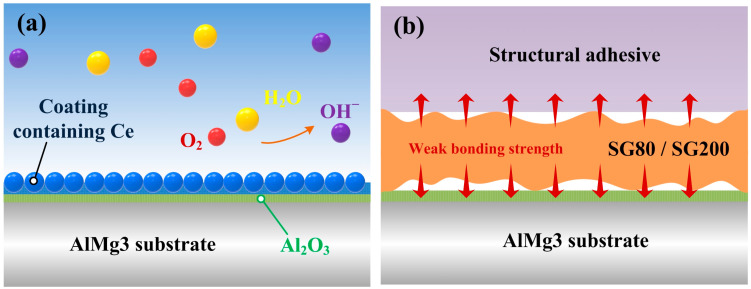
Schematic diagram for the inhibition of coatings containing cerium on the AlMg_3_ surface (**a**) and the weak bonding strength of the sol-gel-solution-treated samples (**b**).

According to the single lap-shear test results shown in Figure 11, the samples treated with sol-gel coatings (S-SG80 and S-SG200) exhibited poor adhesion to the AlMg_3_ substrate. The sol-gel coating was almost entirely removed both before and after aging, which indicates a low adhesive strength for this type of coating on AlMg_3_. This poor adhesion could be due to the insufficient bonding between the sol-gel layer and the aluminum substrate, as sol-gel coatings can sometimes lack strong chemical or mechanical attachments to certain metallic surfaces. Although sol-gel methods, such as those utilizing water glass and citric acid, are often used to create superhydrophobic surfaces or enhance the bond strength with various additives, the specific formulation used here appears to have a limited effectiveness in achieving durable adhesion on AlMg_3_.

Further studies are currently underway to examine how the concentration of the cerium precursor in the solution and the densification temperature impact the anticorrosive performance of the layers prepared by various methods. These investigations aim to optimize the treatment parameters to enhance the corrosion resistance and the durability of the coatings.

## 4. Conclusions

This study presents a straightforward chemical treatment method for enhancing the adhesive bonding properties of an AlMg_3_ alloy, with the objective of improving bond strength and durability through environmentally friendly procedures. The treatment increased the single lap-shear strength of the AlMg_3_ samples. Notably, the H_2_O_2_-treated samples exhibited the highest initial bond strength, reaching 22.5 ± 0.5 MPa and showing a decrease of only 10% (to 18.1 ± 0.2 MPa) after aging under extreme humidity and temperature conditions (70 °C and 100% R.H. for 4 weeks). The surface morphology analysis by SEM revealed uniform and dense cerium oxide films on the treated samples, contributing to cohesive failure modes in lap-shear tests—an indicator of robust adhesive bonds. Electrochemical impedance spectroscopy further confirmed the high corrosion resistance of the treated surfaces, particularly for the samples with cerium chemical layers, which exhibited lower corrosion current densities (e.g., 0.25 μA/cm^2^ for the SG200-treated samples). These results demonstrate that the proposed treatment method is not only effective in enhancing adhesive strength but also promotes long-term durability. Despite the sol-gel coating being excellent at preventing corrosion, it has the disadvantage of having a very weak adherence. Future research could concentrate on using different methods to create sol-gel coatings with stronger bonds. Chemical coatings made with APS or H_2_O_2_ solutions, on the other hand, have the advantages of improved corrosion resistance and bonding strength. This simple, low-cost, and environmentally safe approach is suitable for industrial applications, providing a viable alternative to conventional, multi-step surface treatments that rely on hazardous chemicals. Future research could investigate the performance of this treatment on other aluminum alloys and evaluate its longevity in diverse environmental conditions.

## Figures and Tables

**Table 1:** Chemical composition of AlMg_3_ aluminum alloy used (in wt.%).

Element	Si	Fe	Cu	Cr	Mg	Zn	Al
Content	0.40	0.40	0.10	0.1	2.6–3.6	0.2	Bal.

## Data Availability

The data are contained within this article.
